# Notices, Reviews, &c.

**Published:** 1859-06

**Authors:** 


### NOTICES, REVIEWS, &c.

*Blackwood's Magazine.* $3 a year, re-published by Leonard Scott & Co., New York, abounds this month with ably written articles. The four Reviews, with this, are furnished at ten dollars a year: to wit, Edinburgh, Westminister, North British and London. The Edinburgh for April is of great value.

*The Fireside Monthly* $1 50 a year, single numbers ten cents, H. B. Price, publisher, New York, 32 pp. 8vo., is the only publication of its kind in the civilized world; for, not professedly religious, it is never against religion, wholly original, and excludes fiction; in these respects, we know of no monthly periodical like it, in any land. True men, good and great of every name are invited to write for it, on any practical subject, not sectarian or sectional or partisan, and not over two foolscap manuscript pages at one time.

*The Boston Medical and Surgical Journal*, $3 a year, continues to be the favorite periodical with the educated of the medical profession in New England, circulating also, largely out side of it.

*The Medico Chirurgical Review*, re-published from England, by the Messrs. Wood, of 389 Broadway, is the standard organ of medical literature for England, America and the continent. Quarterly, $3 a year.

*The Scientific American*, $2 a year, New York, is the most valuable scientific publication in this country, and always abounds in articles reliable and practical.

*The Microscopic's Companion*, with the glossary of the principle terms used in microrcopical science, by John M. King, M. D., Cincinnati, Ohio, is announced; we hope to receive an early copy, as the whole subject is of increasing importance in art and science.

Alice Carey, a name beautiful by itself, without suffix or affix, as that of Henry Clay is great alone, has written another book "Pictures of Country Life," characterized by that truthful, life like picturing, which has already placed her name so high in the literary world. There is one character out of place, and we hope that in the subsequent additions which will undoubtedly be called for, it will be modified or re-touched. To represent a minister of the gospel as unfeeling, hard-hearted, unsympathising, is as if a woman was thus represented, and to stand for her sex. Let us all stand by the "ministry of reconciliation," for only by them, as to their teachings can any nation stand, and let woman especially, who owes so much of her exaltation to christian teachings, think, and write, and speak of a clergyman as she would have him be.

"I consider your Journal of Health, as among the reliable things of the day, universally quoted, aud although often attacking popular fallacies, carrying conviction, and commending itself to the common sense of every intelligent man. I think the article on "colds" worth more than money, and would suggest the propeiety of a re-insertion in the fall."—A Correspondent.

"Physical Degeneracy" from the Tribune office, Chicago. Twelve cents; ought to be read and re-read, and pondered on, by every parent who has a child at school. It is an invaluable paper.

